# Determination of Volatiles by Odor Activity Value and Phenolics of cv. Ayvalik Early-Harvest Olive Oil

**DOI:** 10.3390/foods5030046

**Published:** 2016-06-24

**Authors:** Gamze Guclu, Onur Sevindik, Hasim Kelebek, Serkan Selli

**Affiliations:** 1 Faculty of Agriculture, Department of Food Engineering, University of Cukurova, Adana 01330, Turkey; gguclu@cu.edu.tr (G.G.); foodengonursevindik@gmail.com (O.S.); 2 Faculty of Engineering and Natural Sciences, Department of Food Engineering, Adana Science and Technology University, Adana 01110, Turkey; hkelebek@adanabtu.edu.tr

**Keywords:** cv. Ayvalik, olive oil, early-harvest, aroma, OAV, SAFE, phenolic compounds

## Abstract

Ayvalik is an important olive cultivar producing high quality oils in Turkey. In the present study, volatile and phenolic compositions of early-harvest extra virgin olive oil (cv. Ayvalik) were determined. The solvent-assisted flavor evaporation (SAFE) technique was used for the extraction of volatile components. The aromatic extract obtained by SAFE was representative of the olive oil odor. A total of 32 aroma compounds, including alcohols, aldehydes, terpenes, esters, and an acid, were identified in the olive oil. Aldehydes and alcohols were qualitatively and quantitatively the most dominant volatiles in the oil sample. Of these, six volatile components presented odor activity values (OAVs) greater than one, with (*Z*)-3-hexenal (green), hexanal (green-sweet) and nonanal (fatty-pungent) being those with the highest OAVs in olive oil. A total of 14 phenolic compounds were identified and quantified by liquid chromatography combined with a diode array detector and ion spray mass spectrometry. The major phenolic compounds were found as 3,4-DHPEA-EDA, 3,4-DHPEA-EA and *p*-HPEA-EDA.

## 1. Introduction

Olive, a fruit of the olive tree (*Olea europaea* L.), and its oil have always been the key components of the Mediterranean diet for decades due to its high nutritional quality and positive health effects [1]. This fruit has become a representative symbol of Mediterranean civilization as 98% of 900 million olive trees are known to be located in this region which spearheads olive oil production [2].

Olive oil, generally obtained using some mild physical processes (milling, centrifugation, filtering, decanting, etc.) [3], is produced from fresh fruits harvested at their optimum maturity phase [4]. Olive oil is one of the few vegetable oils suitable for direct human consumption without any further refining processes [5,6]. According to data released in 2015, olive oil production in the world reached nearly 2.99 million tons. Turkey meets the demand of olive oil in the world as one of the biggest producers with approximately 143,000 tons [7], especially in the northern and southern parts of the Aegean region, where the majority of olive oil manufacturers in Turkey are located. Ayvalik is one of the most cultivated, middle-sized olive trees with either cylindrical or circular-shaped fruits containing nearly 25% oil with high chemical and organoleptic quality [8,9]. This oil shows high resistance to oxidative degradations due to its rich phenolic and monounsaturated fatty acid profiles [10]. These constituents play a key role in the classification of olive oil into categories such as extra virgin, virgin, and ordinary quality [2,11].

Extra virgin olive oil’s (EVOO) popularity results from its volatile and non-volatile compounds. Non-volatile constituents affect the purity of the olive oil as volatile compounds generate the organoleptic characteristics that play a crucial role in human nutrition and acceptability by consumers [11,12]. EVOO has a redolent and dainty flavor differentiating it from other edible oils [4,13]. Its characteristic aroma is reported to display green and fruity attributes depending on the volatile components, with some of them coming directly from the fruit and some deriving from the degradation of polyunsaturated fatty acids as a result of lipoxygenase (LOX) enzyme activity [13,14]. This unique sensorial characteristic of oil is generated mainly from various compounds that exist in extremely low concentrations such as aldehydes, alcohols, esters, hydrocarbons, ketones, and furans [15,16]. The volatile components of olive oil are mostly formed during the rupture of cell structures through the extraction process, from enzymatic activity and autoxidation of fatty acids [13,16,17,18]. The concentration of these compounds determines the quality, adulteration, rancidity, and even the variety of olive used, and is affected by the olive variety, extraction process, and storage conditions [16,18].

EVOO is unique among the vegetable oils due to the substantial level of phenolic compounds belonging to different families such as benzoic and cinnamic acids, phenyl ethyl alcohols, flavones, secoiridoids, and ligstroside derivatives [16,19,20]. These compounds are beneficial to health, acting as antioxidants and preventing oxidation reactions [20,21]. Olive oil is reported to have a role in prohibiting coronary heart disease and Alzheimer’s disease, providing protection against colon, breast, and ovary cancers and diabetes; these effects are mostly related to the phenolic composition of olive oils [21,22,23]. Phenolic compounds also affect the quality of olive oil, influencing the sensorial properties and nutritional value [24].

In this research, the volatile and phenolic compounds of early-harvest Ayvalik olive oil were investigated. Additionally, the odor activity values (OAV) of volatile compounds and the contribution of these compounds to overall aroma were calculated. The solvent-assisted flavor evaporation (SAFE) method was used for the extraction of volatile compounds from olive oil. Identification and quantification of these compounds were carried out by GC-MS and GC-FID; meanwhile, the phenolic content of olive oil was determined by LC-DAD–ESI-MS/MS.

## 2. Experimental Section

### 2.1. Chemicals

Deionized water (resistivity over 18 MX cm) from a Millipore Q (Millipore Corp., Saint-Quentin, France) water purification system was used in all experiments. Standard aroma compounds such as hexanal, (*Z*)-3-hexenal, (*E*)-2-hexenal, nonanal, (*E*,*E*)-2,4-hexadienal, benzaldehyde, phenylacetaldehyde, 1-penten-3-ol, 1-hexanol, isoamyl alcohol, (*E*)-2-penten-1-ol, (*E*)-3-hexen-1-ol, (*Z*)-3-hexen-1-ol, (*E*)-2-hexen-1-ol, benzyl alcohol, 2-phenylethanol, dl-limonene, α-copaene, β-ocimene, β-sesquiphellandrene, (*E*,*E*)-α-farnesene, and phenolic compounds like caffeic, vanillic, *p*-coumaric, ferulic acids, tyrosol, apigenin and luteolin from Sigma-Aldrich (Steinheim, Germany), and dichloromethane, 4-nonanol, sodium chloride, acetonitrile, formic acid and sodium sulphate were obtained from Merck (Gernsheim, Germany).

### 2.2. Olives and Oil Extraction

Ayvalik cultivar olives were obtained from Mersin Province in the Mediterranean region in Turkey. The general climate of this region is moderate. Early stage olives (having a maturity index nearly 2.5) were collected from selected trees and immediately processed to olive oils. Only sound fruits without any kind of infection or physical damage were used for oil production. The olive fruits were extracted by a three-phase centrifugal decanter (as described by Di Giovacchino et al. [25]), which consists of multiple steps such as leaf removal, washing, crushing, malaxation, and centrifugal extraction, must separate and vertical centrifugal separation of oily liquid [8]. As a result of these processes, fruits fractionate into oil, pomace, and waste water. Fresh olive oils were placed into dark glass bottles and were kept at 4 °C in the refrigerator until analysis.

### 2.3. Standard Chemical Analysis of Olive Oil

Free fatty acids of the samples were calculated following the methods described in Kesen et al. [16] and spectrophotometric parameters (K_232_ and K_270_) by the European Union official methods [26]. The color of the olive oil was measured with a HunterLab ColorFlex colorimeter (Reston, VA, USA). L*, a* and b* results for the sample were recorded using CIE color system. Measurements were made at room temperature. An olive oil sample was placed in a sample cell (6 cm diameter) 2 cm deep and the data were read.

### 2.4. Extraction and Analysis of Volatile Compounds

The extraction of aroma compounds was performed in dichloromethane which is an efficient solvent for the isolation of volatile compounds in fruits and plants [27,28]. The volatiles present in EVOO were extracted using the SAFE unit (GläsblasereiBahr, Manching, Germany) under vacuum (10^−3^ Pa; Vacuubrand DCP 3000, Wertheim, Germany). Before extraction, a 80 mL sample containing 120 mL dichloromethane was put into a 500 mL flask with an addition of 5 µL (40 µg) of internal standard (4-nonanol). The content was stirred at 4 °C for 60 min under nitrogen gas. The organic phase (solvent) was slowly fed into the upper portion of the transfer head. Separation of the mixture occurred when content of the sample was dropped into the round bottom flask that was partially submerged in a warm (40 °C) water bath. Separated volatiles passed through the separation head into a receiving vessel where they condensed and froze because of the sudden drop in temperature. Once the separation was completed, the receiving vessel was removed and allowed to thaw at room temperature for 30 min [27,29,30]. After dehydration by anhydrous sodium sulfate, the pooled aromatic extract was reduced to 5 mL in a Kuderna Danish concentrator (Sigma Aldrich, Saint Louis, MO, USA) fitted with a Snyder column (Supelco, Bellefonte, Pennsylvania, USA) and then to 200 μL under a gentle stream of purified nitrogen. The extracts were subsequently stored at −20 °C in a 2 mL glass vial equipped with Teflon-lined cap until the analysis. Samples were extracted in triplicate and the concentration of aroma compounds was calculated as means.

### 2.5. GC-MS and GC-FID Analysis of Volatile Compounds

The analysis of the volatile components was performed on a GC-MS instrument (Agilent 6890 GC and 5973-Network MSD, Agilent Technologies, Wilmington, DE, USA). Aroma compounds were separated on the DB-Wax column from J & W (30 m length × 0.25 mm i.d. × 0.5 µm thickness, Folsom, California, USA). Three microliters of extract was injected each time in pulsed splitless (40 psi; 0.5 min) mode. The injector and FID detectors were held at 270 and 280 °C, respectively. The carrier gas was helium at 1.5 mL·min^−1^. The GC oven temperature program was first increased from 50 to 200 °C at a rate of 5 °C·min^−1^ and then to 260 °C at 8 °C·min^−1^ with a final hold at 260 °C for 5 min. The same oven temperature programs were used for the MS detector. The mass detector was operated in scan mode with electronic impact ionization (ionization energy of 70 eV) and a mass range of 30–300 amu, and a scan rate of 2.0 scan·s^−1^, used to detect the ions formed. The compounds were identified by comparing their retention index, Wiley 6, and NBS 75 k mass spectra libraries installed in the GC-MS, and of the instrument’s internal library created from the previous laboratory studies. Some of the identifications were confirmed by the injection of the chemical standards into the GC-MS system. Retention indices of the compounds were calculated by using the retention data of the linear alkane (C11–C30) series [28]. The concentrations of volatile compounds were calculated according to the internal standard.

### 2.6. Calculation of Odor Activity Values

The odor activity values (OAVs) were calculated by dividing the concentrations of aroma compounds with their sensory thresholds from the literature [16,31,32]. Only the compounds with an OAV greater than 1 were accepted to contribute individually to the olive oil aroma.

### 2.7. LC-DAD-ESI-MS/MS Analysis of Phenolic Compounds

According to Kelebek et al. [10], a total of 4 g of the oil sample was added to 2 mL of *n*-hexane and 4 mL of a methanol/water (70:30; *v*:*v*) solution in a 10 mL centrifuge tube. After vigorous mixing, they were centrifuged for 15 min at 5500 rpm. The hydro-alcoholic phase was collected, and the hexane phase was re-extracted twice with 2 mL of methanol/water (70:30; *v*:*v*) solution each time. Finally, the hydro-alcoholic fractions were combined, washed with 2 mL of *n*-hexane to remove the residual oil, then concentrated and evaporated under vacuum at 35 °C. The dry extracts were re-suspended in 0.5 mL of a methanol/water (50:50, *v*:*v*) solution and filtered through a 0.2 μm nylon filter (Whatman Inc., Clifton, New Jersey, USA) before being analyzed by LC-ESI-DAD-MS/MS.

An Agilent 1100 HPLC system (Agilent Technologies, Palo Alto, CA, USA) operated by Windows NT-based ChemStation software was utilized; the HPLC equipment was used along with a diode array detector (DAD). The system was comprised of a binary pump, degasser, and auto sampler. The column used was a Phenomenex reversed-phase C-18 column (4.6 mm × 250 mm, 5 μm) (Torrance, California, USA). The mobile phase consisted of two solvents: Solvent A, water/formic acid (99.5:0.5; *v*:*v*) and Solvent B, acetonitrile/solvent A (60:40; *v*:*v*). Under the conditions of 0.5 mL·min^−1^ flow rate with temperature set at 25 °C; isocratic conditions from 0 to 5 min with 0% B; gradient conditions from 0% to 5% B in 20 min; from 5% to 15% B in 18 min; from 15% to 25% B in 14 min; from 25% to 50% B in 31 min; from 50% to 100% B in 3 min; followed by washing and reconditioning of the column, phenolic compounds were eluted. The UV-visible spectra (scanning from 200 nm to 600 nm) were recorded for all peaks. Triplicate analyses were performed for each sample. The identification and assignation of each compound was performed by comparing retention times and UV spectra to authentic standards; and confirmed by an Agilent 6430 LC-MS/MS spectrometer (Santa Clara, CA, USA) equipped with an electrospray ionization source. The electrospray ionization mass spectrometry detection was performed in negative ion mode with the following optimized parameters: capillary temperature 400 °C, N_2_ 12 L·min^−1^; nebulizer pressure, 45 psi [10]. Data gaining was performed using the Multiple Reactions Monitoring (MRM) method that solely monitors specific mass transitions during preset retention times. The standard curves were obtained using the commercial standards of the concentrations normally present in olive oils (approximately 1–100 mg·kg^−1^), obtaining regression coefficients (*r*^2^) above 0.995 in all cases.

## 3. Results and Discussion

### 3.1. Chemical Composition of Olive Oil

The chemical properties of cv. Ayvalik extra virgin olive oil (EVOO) are shown in Table 1. Free fatty acidity values are generally used for quality characteristics. According to the quality indices (free fatty acids and UV absorption parameters, K_232_ and K_270_), the olive oil sample complied with the standard values for “extra virgin olive oil” set by the European Regulation on Olive Oils [26]. Free fatty acid (oleic acid %), K_232_ and K_270_ values were found as 0.614%, 2.00 and 0.155, respectively. Free fatty acid values of EVOO should not be exceed the limit of 0.8% and our result correlates with this standard as reported in other studies carried out with cv. Ayvalik [33,34,35]. Color is a significant property as it highly affects the preference of consumers toward oils [13]. Color values were recorded as L* (33.88), a* (−1.52) and b* (38.03) values. Comparatively, our results are different from the results on cv. Ayvalik olive oil conducted by Kelebek et al. [10]. This result can be caused by the geographical origin of the olives and where the olives were cultivated.

### 3.2. Volatile Composition of the Olive Oil

A number of extraction methods have been used to extract the aroma compounds from various foods. In our study, the SAFE technique was used to extract Ayvalik olive oil volatiles. The obtained aromatic extract by the SAFE method with dichloromethane exhibited the most similar aroma to the original Ayvalik olive oil, when a drop of the aromatic extract was assessed on a cardboard smelling trip (7140 BPSI, Lyas, France). The volatile compounds and linear retention indices determined on the DB-WAX column are displayed in Table 2. A total of 32 compounds were determined in the EVOO of cv. Ayvalik including alcohols, aldehydes, terpenes, esters, and a carboxylic acid. The sum of these compounds was determined as 15,017.7 µg·kg^−1^. Aldehydes were found as quantitatively major components in the overall volatile composition followed by alcohols. The compounds with an OAV higher than 1 are also given in Table 3 and the OAV values were calculated by using the odor threshold values from other studies [16,31,32]. Six compounds were detected at concentrations greater than the corresponding odor threshold values and it is possible these components contribute to the overall aroma of cv. Ayvalik olive oil.

#### 3.2.1. Aldehydes

A total of nine aldehydes were identified in EVOO with a concentration of 6261.8 µg·kg^−1^ accounting for 41.6% of the entire volatile content. Aldehydes are reported to be found in higher amounts than other compounds in olive oil according to the studies [4,5,16,36]. Additionally, aldehydes constituted the majority of total volatiles in cv. Ayvalik oil. First, (*E*)*-*2-Hexenal was assessed as the most abundant compound with a concentration of 2540 µg·kg^−1^. This component is formed through the LOX pathway and isomerization of (*Z*)*-*3-hexenal (1137 µg·kg^−1^) which is the third major aldehyde after hexanal (1777 µg·kg^−1^). Then (*Z*)*-*3-Hexenal and hexanal have higher proportions among the aldehydes and are produced as a result of the degradation of 13-hydroperoxides by the hydroperoxide lyase (HPL) enzyme [37]. These aldehydes are present in almost all olive oil cultivars [1,6,33,38]. Kesen et al. [16] reported to identify (*E*)*-*2-hexenal as the ascendant compound followed by hexanal in extra virgin olive oil. Similarly, Romero et al. [39] determined (*E*)*-*2-hexenal as the most abundant compound in Chilean olive oil. In addition, (*E*)*-*2-hexenal is stated to be one of the main participators in the overall aroma of olive oils, existing in high amounts [40]. The odor threshold values of aldehydes are generally lower than those of volatile compounds; thus, they have an important effect on the total flavor of olive oils. As displayed in Table 3, aldehydes make the most significant contribution to the aroma profile of cv. Ayvalik oil.

A total of four aldehydes were identified as having the highest OAVs including hexanal (18.8), nonanal (7.3), (*E*)-3-hexenal (379.2) and (*E*)-2-hexenal (6). Within aldehydes, (*Z*)-3-hexenal has the majority with an OAV followed by hexanal and nonanal. Then (*Z*)-3-Hexenal and hexanal are known to have green and sweet odors, having a positive effect on aroma, produced by the LOX pathway; moreover, hexanal may also be formed through the oxidation of linoleic acid [34]. In addition, Morales et al. [41] reported that the hexanal/nonanal ratio indicates the oxidation status of olive oils. This ratio should be higher than two for the oils not being oxidized and our result matches with this information as the ratio was found to be 4.9.

#### 3.2.2. Alcohols

Alcohols were found as the second main group of the volatile compounds in the sample. The total amount of these compounds equaled 6190.8 µg·kg^−1^. C6 alcohols such as (*Z*)-3-hexen-1-ol, (*E*)-2-hexen-1-ol and 1-hexanol are present in abundant amounts in oil and they provide green-grassy odors. As Sarolic et al. [18] stated, variable amounts of 1-hexanol are formed from the degradation of linoleic acid; (*E*)-2-hexen-1-ol and (*Z*)-3*-*hexen-1-ol are developed as a result of the enzymatic degradation of linolenic acid. Toker et al. [34] also determined these compounds in cv. Ayvalik olive oil and (*Z*)-3-hexen-1-ol is reported to have the highest concentration among aldehydes, similar to our study. Additionally, C6 alcohols are determined in Spanish [42] and Tunisian [13] cultivars in other studies. In terms of OAVs, 1-hexanol (fruity, green) and 3-penten-2-ol (perfumery, woody) are the main contributors to the olive oil aroma. Additionally, 1-Hexanol is determined as the aroma-active alcohol compound in cv. Ayvalik olive oil by Kesen et al. [35] and thus its effect on aroma is higher than with other alcohols.

#### 3.2.3. Terpenes

Terpenes including β-ocimene, α-copaene, β-sesquiphellandrene, (*E*)-β-farnesene, zingiberene, and (*E*,*E*)-α-farnesene were detected in olive oil with a total amount of 2331.2 µg·kg^−1^. Terpenes β-sesquiphellandrene (749 µg·kg^−1^), (*E*,*E*)-α-farnesene (545 µg·kg^−1^) and (*E*)-β-farnesene (466 µg·kg^−1^) have the highest concentrations among all the terpenes. These compounds were also detected by Kesen et al. [35] in another study on cv. Ayvalik EVOO, by Kosma et al. [38] in five less well-known Greek cultivar EVOOs and by Issaoui et al. [43] in Tunisian cv. olive oil. Additionally, Kaftan and Elmaci [36] also determined β-sesquiphellandrene in cv. Ayvalik olive oil. Besides, as Bubola et al. [44] implied, these terpenoid hydrocarbons show a difference in terms of amount and variety according to the cultivars of olive oil [38,45]. Kesen et al. [35] determined the terpenoid compounds such as α-copaene (sweet, fruity), zingiberene (floral), (*E*,*E*)-α-farnesene (floral, herb) and β-sesquiphellandrene (floral) as the aroma-active components in olive oil extracts. In the study performed by Kosma et al. [38], terpenes are reported to display differences among five EVOO cultivars. This result shows that terpenoid compounds are highly dependent on the variety of olives used.

#### 3.2.4. Esters

Esters are responsible for the fruity and flowery aroma in many fruits [16]. Three esters were determined in the oil, with a total concentration of 236.4 µg·kg^−1^, including linalyl propionate, hexyl acetate, and methyl salicylate. Hexyl acetate was found abundantly with a concentration of 164 µg·kg^−1^ in the group of esters. This compound is known to be produced in the LOX pathway with the activity of the alcohol acyltranferase (AAT) enzyme which carries out the formation of acetate esters [31,37,38]. Hexyl acetate is known to have sweet, floral, or fruity notes in olive oils [6,31,33]. Kesen et al. [35] have also determined this compound in Ayvalik olive oil in addition to Memecik and Gemlik cultivars. In the extant literature, this component is detected in Spanish Picual and Arbequina oils [40], Tunisian oils [43], Chilean EVOOs [39] and Greek EVOOs [38].

Another group of volatiles identified in Ayvalik oil was carboxylic acids. In this group, only butanoic acid was determined with a minor concentration of 1.75 µg·kg^−1^. It is also detected in other olive oil varieties and gives a fusty or rancid odor at high concentrations [46].

### 3.3. Phenolic Composition of *cv.* Ayvalik Olive Oil

A sum of 14 phenolic compounds consisting of phenolic alcohols such as hydroxytyrosol (3,4-DHPEA), tyrosol (*p*-HPEA), phenolic acids such as ferulic, vanillic, *p*-coumaric and caffeic acids, secoiridoids including hydroxytyrosol acetate (3,4-DHPEA-AC), dialdehydic form of elenolic acid linked to hydroxytyrosol (3,4-DHPEA-EDA), oleocanthal (*p*-HPEA-EDA), oleuropein aglycon (3,4-DHPEA-EA), ligstroside aglycon (*p*-HPEA-EA), and flavonoids including luteloin, apigenin and diosmetin were determined in the composition of the oil. These compounds with their chemical structures were also mentioned in the report by Servili et al. as the phenolics of virgin olive oil [47]. Table 4 shows the identified compounds with information such as retention time, λ_max_ in the UV region, molecular ions, main fragment ions in MS/MS, and tentative identification. The total amount of phenolic compounds was determined as 228 mg·kg^−1^. It is reported in many research projects that phenolic compounds and their concentrations may show a difference according to the olive variety and ripening stage, climacteric factors, extraction methods, and storage conditions [16,48,49]. Kelebek et al. [10] determined the phenolic content of cv. Ayvalik olive oil obtained from the northern Aegean region of Turkey with an amount of 93.2 mg·kg^−1^ in 2011. They also reported that there are significant differences in phenolic contents among olive oil varieties affecting the sensory characteristics and stability of the oil [10]. As Peres et al. [17] stated, high phenolic content represents fruits which were superior in quality and no damaged olives were used. This statement also supports our results.

In terms of the individual analysis of phenolic compounds, 3,4-DHPEA-EDA (123.11 mg·kg^−1^) has the majority in overall phenolics, followed by oleuropein aglycon (54.40 mg·kg^−1^) and *p*-HPEA-EDA (20.02 mg·kg^−1^). As seen in Table 4, secoiridoid (SEC) derivatives are the most abundant phenolic compounds in cv. Ayvalik oil. Then 3,4-DHPEA-EDA and 3,4-DHPEA-EA are formed as a result of the esterification of hydroxytyrosol, while *p*-HPEA-EA is an ester of tyrosol [50]. Phenolic compounds of EVOO are known to have a significant effect in sensory properties generally related to bitterness and raciness. Peres et al. [17] indicated that 3,4-DHPEA-EA, *p*-HPEA-EA, 3,4-DHPEA-EDA and *p*-HPEA-EDA are associated with these properties as oleocanthal (*p*-HPEA-EDA) has higher effects on sensory receptors. They also reported that 3,4-DHPEA-EDA had the majority among *p-*HPEA and 3,4-DHPEA derivatives with an amount of 247 mg·kg^−1^ showing similarity in our results. It is also reported that *p*-HPEA-EDA and 3,4-DHPEA-EA were the most abundant SECs in the oils following 3,4-DHPEA-EDA. Oxidation is known to affect the SECs of oil as the amount of SECs decreases and simple phenols such as tyrosol and hydroxytyrosol, elenolic acid, oxidized forms of elenolic acid and oxidized forms of secoiridoids are formed during storage [51]. SECs are especially important in terms of the health benefits they provide in olive oils, such as the inhibition of phospholipid oxidation and providing anti-inflammatory activity [50].

Flavonoids are the second effective group with a total amount of 12.07 mg·kg^−1^ following SECs in olive oil. Luteolin, apigenin and diosmetin were determined among the flavonoids with concentrations of 6.16, 3.92 and 2 mg·kg^−1^, respectively. These flavones are also found in Greek [51] and Turkish [10] olive oils. Apigenin is dictated as the substrate for the formation of luteolin in the flavonoids pathway by activity of a hydroxylase enzyme, and diosmetin is known to be a methoxyderivative of luteolin [50].

In regard to phenolic alcohols, *p*-HPEA (tyrosol) and 3,4-DHPEA (hydroxytyrosol) were found in olive oil. The total amount of this group of phenolics was determined as 8.47 mg·kg^−1^ and within these compounds, 3,4-DHPEA was found as the main phenolic alcohol in oil with an amount of 5.16 mg·kg^−1^. The *p*-HPEA and 3,4-DHPEA were described in Ayvalik and other Turkish olive oil cultivars [10,16]. In the extant literature, these two phenolic alcohols were determined in VOOs from olive cultivars grown in Greece [51] and Spain [52]. Hydroxytyrosol has importance in the antioxidant capability of oil. This compound is also reported to have a role in the prevention of cancer, cardiovascular diseases and diabetes [50].

Hydroxycinnamic acids were identified in olive oil as phenolic acids including ferulic (0.72 mg·kg^−1^), vanillic (0.04 mg·kg^−1^), *p-*coumaric (3.38 mg·kg^−1^) and caffeic acids (0.24 mg·kg^−1^). Within phenolic acids, *p*-coumaric acid was detected in comparatively higher amounts and ferulic acid was found the second most abundant phenolic acid in EVOO. These phenolic acids were also detected, except ferulic acid, in Portuguese olive oil [17] and seven Spanish monovarietal VOOs [50].

## 4. Conclusions

In the present study, a remarkable amount of data about volatiles, OAVs and phenolic compounds of early-harvest cv. Ayvalik EVOO was obtained. Aldehydes were found as the most abundant and significant volatiles followed by alcohols in olive oil. Furthermore, (*E*)-2-Hexenal has been found as the major aldehyde in the sample. With regards to aroma contribution to olive oil, six compounds were leading based on OAVs: (*Z*)-3-Hexenal has shown the greatest OAV followed by hexanal and nonanal. According to LC-DAD-ESI-MS/MS results, 3,4-DHPEA-EDA was found as the prominent phenolic, followed by 3,4-DHPEA-EA and *p*-HPEA-EDA. Finally, all these results provide a beneficial contribution in understanding the volatile and phenolic compositions of early-harvest cv. Ayvalik oil.

## Figures and Tables

**Table 1:** General properties of olive oil ^a^.

Free Fatty Acid (Oleic Acid %)	0.61 ± 0.02
K_232_	2.00 ± 0.00
K_270_	0.15 ± 0.00
L*	33.88 ± 1.02
a*	−1.52 ± 0.04
b*	38.03 ± 0.06

^a^ Results are mean of three replications ± standard deviation.

**Table 2:** Volatile composition of cv. Ayvalik extra virgin olive oil.

No.	LRI ^a^	Compounds	Concentration ^b^	Identification ^c^
*Aldehydes*				
1	1074	Hexanal	1777	LRI, MS, Std
2	1182	3-Methyl-2-butanal	178	LRI, MS, tent
3	1211	(*Z*)-3-Hexenal	1137	LRI, MS, Std
4	1220	(*E*)-2-Hexenal	2540	LRI, MS, Std
5	1384	Nonanal	364	LRI, MS, Std
6	1485	(*E*,*E*)-2,4-Hexadienal	65.8	LRI, MS, Std
7	1527	Benzaldehyde	79.7	LRI, MS, Std
8	1642	Phenylacetaldehyde	57.2	LRI, MS, Std
9	1759	(*E*,*E*)-2,4-Heptadienal	63.1	LRI, MS, Std
		Total	6261.8	
*Alcohols*				
10	1157	1-Penten-3-ol	230	LRI, MS, Std
11	1177	3-Penten-2-ol	849	LRI, MS, tent
12	1221	Isoamyl alcohol	344	LRI, MS, Std
13	1298	2-Hexanol	227	LRI, MS, tent
14	1318	(*E*)-2-Penten-1-ol	89	LRI, MS, Std
15	1350	1-Hexanol	747	LRI, MS, Std
16	1371	(*E*)-3-Hexen-1-ol	124	LRI, MS, Std
17	1378	(*Z*)-3-Hexen-1-ol	1955	LRI, MS, Std
18	1412	(*E*)-2-Hexen-1-ol	1036	LRI, MS, Std
19	1861	Benzyl alcohol	41.5	LRI, MS, Std
20	1925	2-Phenylethanol	119	LRI, MS, Std
21	2107	2-Phenoxyethanol	425	LRI, MS, tent
		Total	6186.5	
*Terpenes*				
22	1186	dl-Limonene	11,2	LRI, MS, Std
23	1250	β-Ocimene	233	LRI, MS, Std
24	1440	α-Copaene	103	LRI, MS, Std
25	1634	β-Sesquiphellandrene	749	LRI, MS, Std
26	1666	(*E*)-β-Farnesene	466	LRI, MS, tent
27	1702	Zingiberene	224	LRI, MS, tent
28	1719	(*E*,*E*)-α-Farnesene	545	LRI, MS, Std
		Total	2331.2	
*Esters*				
29	1279	Hexyl acetate	164	LRI, MS, Std
30	1696	Linalyl propionate	12.1	LRI, MS, Std
31	1747	Methyl salicylate	60.3	LRI, MS, Std
		Total	236.4	
*Carb. Acid*				
32	1637	Butanoic acid	1.75	LRI, MS, Std
		Total	1.75	
*General Total*			15,017.7	

^a^ LRI: Linear retention index calculated on DB-WAX capillary column; ^b^ Concentration: Results are the means of three repetitions as µg·kg^−1^; ^c^ Identification: Methods of identification; LRI (linear retention index), MS tent, (tentatively identified by MS), Std (chemical standard); When only MS or LRI is available for the identification of a compound, it must be considered as an attempt of identification. Standard deviation of all aroma compounds was below 10%.

**Table 3:** Odor thresholds and odor activity values (OAVs) of potent volatile compounds in Ayvalik olive oil.

No.	Compounds	Odor Threshold Value	Odor Activity Value	Odor Descriptions
1	Hexanal	75 [31]	18.8	Green apple, grassy
2	(*Z*)-3-Hexenal	3 [32]	379	Green
3	(*E*)-2-Hexenal	424 [31]	6	Green, apple-like
4	Nonanal	150 [31]	7.3	Fatty, waxy, pungent
5	3-Penten-2-ol	400 [31]	2.12	Perfumery, woody
6	1-Hexanol	400 [16]	1.9	Fruity, green

Odor threshold values (µg·kg^−1^) were taken from the literature (Kesen et al. [16], Kalua et al. [31], Aparicio et al. [32]). OAVs were calculated by dividing the concentration by the odor thresholds.

**Table 4:** Main phenolic compounds identified in olive oil extract by HPLC-DAD-ESI-MS/MS including: retention time (RT), molecular formula (MF), λ_max_, LC-MS/MS parameters and MRM transitions.

No.	Compounds	RT (min)	MF	λ (nm)	Fragmentor (v)	Precursor Ion	Collision Energy (v)	Quantitative Transition (m/z)	Ayvalik EVOO (mg·kg^−1^)
	*Phenolic alcohols (PAL)*								
1	3,4-DHPEA	13.63	C_8_H_10_O_3_	235.280	115	153	15	153 > 123	5.16 ± 0.1
2	*p*-HPEA ^x^	17.28	C_8_H_10_O_2_	237.275	115	137	15	137 > 119	3.05 ± 0.17
	*Total PAL*								8.47 ± 0.27
	*Phenolic acids (PA)*								
3	Ferulic acid^x^	37.05	C_10_H_10_O_4_	323.293	90	193	15	193 > 134	0.72 ± 0.01
4	Vanillic acid ^x^	36.70	C_8_H_8_O_4_	258.293	90	167	15	167 > 123	0.04 ± 0.00
5	*p*-coumaricacid ^x^	29.06	C_9_H_8_O_3_	236.310	90	163	15	163 > 119	3.38 ± 0.13
6	Caffeic acid ^x^	31.2	C_9_H_8_O_4_	325.000	90	179	15	179 > 135	0.24 ± 0.01
	*Total PA*								4.38 ± 0.15
	*Secoiridoids (SEC)*								
7	3,4-DHPEA-AC	32.48	C_10_H_12_O_4_	252.275	115	195	15	195 > 59	1.24 ± 0.05
8	3,4-DHPEA-EDA	39.49	C_17_H_20_O_6_	246.275	135	319	25	319 > 59	126.42 ± 3.31
9	*p*-HPEA-EDA	46.01	C_17_H_20_O_5_	-	115	303	25	303 > 59	19.28 ± 0.74
10	3,4-DHPEA-EA	47.44	C_19_H_22_O_8_	243.281	115	377	25	377 > 275	56.55 ± 2.15
11	*p*-HPEA-EA	54.13	C_19_H_22_O_7_	246.275	115	361	25	361 > 291	4.21 ± 0.16
	*Total SEC*								207.68 ± 4.52
	*Flavonoids (FLA)*								
12	Luteolin ^x^	43.00	C_15_H_10_O_6_	254.350	135	285	35	285 > 133	5.98 ± 0.19
13	Apigenin ^x^	50.32	C_15_H_10_O_5_	234.338	135	269	35	269 > 117	3.78 ± 0.15
14	Diosmetin ^x^	51.79	C_16_H_12_O_6_	-	135	299	25	299 > 284	1.93 ± 0.08
	*Total FLA*								11.67 ± 0.4

^x^ Identification confirmed by comparison with standards. 3,4-DHPEA: Hydroxytyrosol; *p*-HPEA: Tyrosol; 3,4-DHPEA-AC: Hydroxytyrosol acetate; *p*-HPEA-EA: Ligstroside aglycon; 3,4-DHPEA-EA: Oleuropein aglycon; 3,4-DHPEA-EDA: Dialdehydic form of elenolic acid linked to hydroxytyrosol.
